# Supplementary material for: Are carbon emissions trading and green financial instruments synergistic? -Comprehensive quantitative research based on content analysis
Source: PLoS One. 2024 Mar 7;19(3):e0298601. doi: 10.1371/journal.pone.0298601 (PMC10919601; doi:10.1371/journal.pone.0298601)
Supplement: S1 Data — (ZIP) [file pone.0298601.s001.zip › Data/地级市Z_F工作报告.zip!/╣π╬≈/║╪╓▌╩╨/└┤╫╘╣1⁄2╓┌║┼í╛┬φ┐╦╩2╛▌═°í┐_2003.docx]

各位代表：

　　现在，我代表贺州市人民政府，向大会报告工作，请予审议，并请市政协各位委员和其他同志提出意见。

2002年工作的回顾

　　去年，在邓小平理论和“三个代表”重要思想指引下，在中央、自治区的正确领导和市委的直接领导下，在全市各族人民的大力支持下，我市紧紧把握撤地设市、加快发展这个主题，积极应对加入WTO所带来的机遇和挑战，加快推进实施“十五”计划和西部大开发，大力推进经济结构战略性调整，加快工业化、城镇化步伐，深化改革，扩大开放，促进发展，保持稳定，较好地完成了年初确定的各项目标任务，经济和社会发展取得了新的业绩。

　　国民经济稳步增长，经济结构逐步优化，经济效益进一步好转。初步统计，全市完成国内生产总值108.98亿元，比上年增长8%；全社会固定资产投资完成12.51亿元，增长10.6%；外贸进出口总额9008万美元，增长32.5%；城乡居民消费价格总水平下降1.8%。农业结构调整进一步优化，优质和特色农产品加快发展，全市优质水稻比例达72.7%，水果、蔬菜良种覆盖率分别达88%和90.1%；新种速生丰产林7.2万亩，完成水果低改8万亩；养殖业加快发展，涌现了三金畜牧水产、金犊种畜、富川大自然奶业等一批养殖业龙头企业，畜牧水产业已占农业总产值的38.4%。工业结构调整力度加大，全市规模以上工业企业完成工业增加值7.13亿元，全年盈亏相抵后实现利润3200万元；电力、林化、医药等优势产业发展加快，一批优势企业通过技术改造不断发展壮大；工业园区建设进展顺利；乡镇企业结构调整取得新进展。服务业保持较快增长，对经济增长的贡献率达到12.7%，尤其是旅游业发展迅猛，全年接待海内外游客155万人次，旅游收入6.2亿元，比上年增长53.28%。非公有制经济得到新的发展，在扩大就业、增加财政收入等方面发挥了重要作用。城镇化进程加快，贺州广场、灵峰广场、星光大道、江滨大道、鞍山路、平安路等一批基础设施项目相继竣工，龟石供水工程、桂梧高速公路贺州段等重点项目已开工建设，贺州市作为中心城市功能有了一定改善，小城镇建设水平进一步提高,三县一区在全区市容市貌“南珠杯”竞赛中实现了满堂红。财政运行正常，全市财政收入完成6.26亿元,下降6.3%，但剔除钟山和富川两烟厂关闭减收3318万元、执行西部大开发优惠政策所得税税率下调减收3191万元、遭受特大洪涝灾害农业税减收846万元等因素，则比上年增加6198万元，增长9.3%；财政一般预算支出完成10.25亿元，增长10.3%，支出结构进一步优化。面对比较严峻的财政形势，我们切实加强财政管理，深化财政改革，在自治区的大力支持下，保证了全市干部职工工资按时发放，确保了各项法定支出稳定增长。金融秩序稳定，服务水平明显提高。

　　改革开放不断深入，经济发展环境进一步改善。我们坚持以市场为导向，深化各项改革。国有企业改革进一步深化，梧州松脂、通盛公司等一批骨干企业已建立起比较规范的现代企业制度；放开搞活中小企业取得新进展，一批中小企业通过改制、承包、租赁等措施重新焕发出生机；企业内部改革整顿继续深化，管理水平有新的提高。社会保障制度进一步完善，养老保险、失业保险、医疗保险覆盖面扩大，国有企业下岗职工和离退休人员的基本生活费按时足额发放，对城镇低保对象实行了应保尽保。农村改革不断深入，粮食流通体制改革稳步推进。结合撤地设市，深化人事制度改革，转变政府职能，尤其在减少审批事项、简化审批程序、规范审批管理方面取得了新成绩。财税、金融、投融资等管理体制改革按自治区的统一部署全面推进。整顿和规范市场经济秩序取得了阶段性成果，关停并转了一批小钢厂、小矿山，强化了安全生产责任制。对外开放取得新突破，全年外贸出口7734.5万美元，增长31.3%;全市开展了“投资环境建设年”活动，投资环境有所改善，促进了招商引资工作，全市新批外商投资项目13个，实际利用外资1807万美元，增长10.51％。

　　科技、教育加快发展，社会事业全面进步。实施科技创新计划成效显著，科技与经济结合取得新进展。各类教育持续发展，教学条件进一步改善，普通高考取得好成绩，“两基”教育进一步巩固。文化艺术、广播电视事业健康发展，《卖桃》等一批优秀作品获得了全国、全区性奖项。人口出生继续稳定低生育水平，出生率 8.37‰，自然增长率5.35‰,计生工作整体水平有较大幅度提高。县（区）、乡（镇）、村三级医疗预防保健网进一步完善，重点传染病的防治和控制得到了加强，城乡居民医疗保健水平进一步提高。全民健身活动广泛开展，竞技体育取得良好成绩。国土资源开发利用和保护工作力度加大，环境保护得到加强，全市工业污染排放达标率95%。人事、机构编制、工商管理、技术监督、审计、统计、地矿、农机、气象、档案、修志等各项工作在经济建设和社会发展中作出了积极贡献。民族宗教、老龄、妇女儿童和残疾人等事业取得新的成绩。

　　城乡居民收入增加，人民生活继续改善。去年，在遭受特大洪涝灾害的情况下，农民人均纯收入预计达1792元，增长3.3%。城镇居民人均可支配收入7030元，增长17.22%。全社会消费品零售总额增长10%，居民消费价格总水平稳中略降。扶贫开发继续推进，全市共投入扶贫资金1670万元，建成一批乡村道路、沼气池、人畜饮水等项目，贫困村的生产、生活条件得到进一步改善，解决了农村贫困户5100户25400人的温饱问题。各县（区）的一大批公益性项目建成并投入使用，城乡居民居住质量进一步提高 。

　　精神文明建设取得新的成绩，民主法制建设进一步加强。通过学习江总书记的“三个代表”的重要思想和“5•31” 讲话以及党的十六大精神，干部群众的思想政治素质进一步提高。以创建桂东文明绿洲为重点的精神文明创建活动广泛深入开展。依法治贺全面推进，依法行政状况进一步改善。基层政权建设继续加强，村民自治整体水平得到提高。廉政建设和反腐败斗争深入开展，政府自身建设得到加强。“严打”专项斗争取得新成效，与“法轮功”邪教组织斗争深入开展，农村“三大纠纷”调处工作取得新成绩，社会治安有所好转。优抚安置和双拥工作力度加大，国防教育和民兵预备役工作进一步加强。全市呈现出经济繁荣、社会稳定、人民安居乐业的良好态势。

　　在过去的一年里，我市全面建设小康社会，加快推进社会主义现代化建设迈出了坚实的步伐。这是在中央、自治区和市委的正确领导下，全市各族人民同心同德、团结奋斗的结果。在此，我谨代表市人民政府向全市各族人民，向驻贺部队官兵，向各民主党派、工商联、人民团体以及所有关心帮助我市改革和发展的各界人士，致以衷心的感谢和崇高的敬意！

各位代表，在总结去年工作成绩的同时，我们也要清醒地看到前进中还有不少困难和问题，主要有：经济总量偏小，经济结构不合理，产业的竞争力不强；在工业结构调整中，新的增长因素不多，一些企业关闭破产后没有替代产业，导致工业增长减缓；农民收入增长不快，部分城镇居民生活困难，就业压力大；财政收支矛盾突出，财源结构单一状况依然没有改变；城市化水平不高，城市规划、建设和管理水平还需进一步提高；一些地方的社会治安状况仍不令人满意，等等。这些问题已引起我们的高度重视，我们将采取积极措施，努力加以解决。

2003年经济社会发展的主要任务

　　今年是全面贯彻落实党的十六大精神的第一年，是本届政府成立后的第一年，也是我市实施“十五”计划和西部大开发战略，全面建设小康社会的关键一年，搞好今年的工作，具有十分重要的意义。在新的一年里，我们要以邓小平理论和“三个代表”重要思想为指导，认真贯彻党的十六大精神，坚持以发展为第一要务，抓住国家实施西部大开发，继续实施积极的财政政策和稳健的货币政策的机遇，以桂梧高速公路、洛湛铁路等重点项目开工建设为契机，着力抓好城镇经济、工业园区经济、生态旅游经济、农业企业经济，促进经济结构战略性调整，确保财政增长，城乡居民增收。切实加强民主法制建设和精神文明建设，维护社会稳定，促进社会全面进步。

根据上述总体要求，综合考虑多方面因素，建议今年我市国民经济和社会发展的主要目标确定为：国内生产总值增长10%，财政收入增长8%，全社会固定资产投资增长30%，全社会消费品零售总额增长12%，外贸出口增长10%，实际利用外资增长10%，农民人均纯收入增长5%，人口自然增长率控制在8‰以内。

　　一、进一步调整农业和农村经济结构，促进农业增效，农民增收

农业、农村和农民问题是关系全市经济社会发展的全局性问题。我市的基础在农业，优势也在农业，必须始终把农业放在突出位置，确保农业增产，农民增收，农村稳定。为适应农业发展和农产品市场格局的新变化以及加入WTO的要求，今年农业和农村工作总的要面向国内外市场尤其是粤港澳市场，围绕优化农业和农村经济结构，大力推进农业产业化经营，提高农产品的市场竞争力来展开，努力促进传统农业向现代农业转变，增加农民收入。

　　大力推进农业结构调整。要进一步调整种植业结构，在优化粮食品种结构，确保粮食生产安全的基础上，合理调整粮经作物种植比例，大力发展效益好的经济作物。要切实搞好农业产品结构调整，着力推广优新品种，对现有的无公害产品、绿色食品和有机食品要扩大生产规模，努力提高农产品优质品率。调整农产品布局，引导特色农作物向优势产区集中，进一步巩固和发展优质稻、蔬菜、马蹄、茶叶、烟叶、桑蚕、红瓜子等农业基地，脐橙、沙田柚、青梅、三华李、龙眼等名优水果基地，以及瘦肉型猪、楼来牛、楼黄杂交牛、奶水牛、银鱼等畜牧水产基地。发挥我市作为全区重要林区的优势，充分利用好国家退耕还林的政策，在搞好短轮伐期速丰林的同时，重点抓好阔叶树速丰林和优质松脂林的建设。要积极推进农业标准化生产，加快农产品质量安全检测体系和认证体系建设，筹建贺州市农产品质量检测中心和饲料、兽药、动物产品检测中心，切实加强农副产品的质量卫生安全监督管理。办好农业示范园（区），发展生态观光农业。

　　努力提高农业产业化经营水平。要把发展龙头企业作为促进农业产业化的重点，在扶持壮大现有龙头企业的同时，再发展一批林业、水果、蔬菜、畜牧、水产等龙头企业，以此增强对农民的带动力和对农村经济的辐射力。要鼓励龙头企业以发展“订单农业”等多种形式与农户联合，实行产供销一条龙、贸工农一体化。加快农产品市场体系建设，不断扶持壮大农产品营销大户和经纪人队伍，进一步搞活农产品的流通。积极推进农民专业合作经济组织建设，建设好市、县（区）13个专业协会，提高农民的组织化程度，促进农业产业化经营。

　　加快以科技、信息服务为重点的农业社会化服务体系建设。重点抓好县（区）、乡（镇）农技推广网络建设，稳定队伍，转变机制，提高效率，增强服务功能。切实加强对农民的科技培训，提高广大农民的科技文化素质和致富本领。搞好农业信息化“进村入户”工程的实施，进一步完善市、县（区）农业信息网络功能，及时准确地向农民提供价格信息、生产信息、气象信息以及中长期市场预测分析，帮助农民按照市场需求进行生产和经营。

　　加强农业和农村基础设施建设。要抓住今年国债资金向农村倾斜的机遇，积极争取国债资金并努力增加财政投入和引导社会资金的投入，进一步改善农村生产生活条件和教育设施。继续抓好以水利为重点的农业基础设施建设，突出抓好龟石灌区续建配套与节水改造工程、昭平县白坭冲小流域治理工程的实施，建立龟石、合面狮两座水库的水情自动测报系统和洪水调度系统。配合“西电东送”工程的实施，做好上程、下福、巴江口、金牛坪、水晶塘等中小型水电站的项目前期和部分项目开工建设工作。今年把八步区建设成为全国电气化县（区）。新建20处饮水工程，解决3万人饮水困难问题。以新建13500座沼气池为重点，抓好农村生态能源建设。抓紧实施农网改造二期工程，启动市区和各县城电网改造工程。实施新阶段的扶贫开发规划，加大扶贫资金的投入，进一步加强贫困村水、电、路、教育、卫生、广播电视等基础设施建设。

　　稳定农村政策。要坚持“多予、少取、放活”的方针，全面落实党在农村的各项政策。继续抓好农村土地延包的后续完善工作，赋予农民长期而有保障的土地使用权。有条件的地方可按照依法、自愿、有偿的原则进行土地承包经营权流转，逐步发展规模经营。要认真贯彻执行“一项制度、八个禁止”的政策，切实减轻农民负担，严肃查处加重农民负担的恶性案件，确保社会稳定。

　　二、提高工业发展水平，积极推进新型工业化

　　加快发展工业，提高工业化水平，是我市今后相当长一个时期的战略重点。政府工作必须紧紧抓住这个重点不放松。今年,全市工业经济的发展,要以推进新型工业化进程为目标, 重点围绕改革搞活一批,技改壮大一批,招商引进一批，筛选储备一批来展开，努力扩大工业经济总量，提高工业质量。

深化改革，加快工业结构的调整，进一步把国有企业搞好搞活。以产权制度改革为核心，对条件成熟的国有骨干企业，促其尽快建立现代企业制度，完善公司法人治理结构，特别要帮助梧州松脂股份有限公司年内完成上市申报工作。进一步搞好国有中小企业，采取主辅业分离、减员增效、减轻债务负担等有力措施，增强主业竞争力，吸引社会投资，通过改组、联合、兼并、租赁、股份合作制、出售等形式放开搞活。协助搞好平桂矿务局的破产工作，支持重组企业尽快生产；做好钟山、富川烟厂破产的后续工作，新上项目力争6月底前投产。要把改革、改组与科学管理结合起来，切实加强企业内部管理，增强企业活力。

　　加快企业技术改造步伐，扶持现有优势企业做强做大。继续增加企业技术改造投入，提高企业的技术装备水平和产品创新能力。技术改造要围绕开发新产品进行，鼓励企业通过引进先进设备进行产品创新，增加科技含量，加速名牌、优势、特色产品上规模、上档次。要重点支持桂东电子有限公司中高压铝箔技改及电子电容器项目、梧州松脂股份有限公司年产1500吨合成芳樟醇项目、灵峰药业年产50万箱中成药技改项目、平桂飞碟公司30万吨回转窑水泥工程技改项目和伟达矿业年产5000吨无铅焊锡项目。这些技改项目年内要投产或部分投产。

　　以工业园区建设为突破口，加大招商引资力度，以开放促进工业的快速发展。要进一步加强对旺高、信都、西湾工业区和八步高科技园的建设，采取政府引导、多元投入、滚动开发的方式，筹措资金，高起点、高标准地搞好园区的规划，加快园区内交通、供电、供水、通信和其他配套服务设施建设，使开发区成为政策优惠、环境宽松、设施完善、服务周到的吸引外商投资的一个示范窗口。旺高工业区，要重点发展食品、化工、医药、纺织等工业；西湾工业区，要重点发展有色金属工业、矿产业以及矿产业的替代产业；信都工业区，要重点承接闽、粤、港、澳的劳动密集型产业；八步高科技工业园，要重点发展高科技产业，形成电子元器件等新兴产业基地。经过几年的努力，使工业园区成为我市工业的重要依托。与此同时，要加强对现有乡镇企业示范区的指导和扶持，继续抓好新的自治区“乡镇企业示范区”的创建申报工作。

　　以项目为中心，培育和发展新的工业经济增长点。加快培育和发展一批新的工业骨干企业，是我们必须长期坚持的一条重要方针。今年，要设立项目办公室，切实加强事关全局的重大项目的前期工作。突出抓好贺达纸业年产15万吨高级文化纸的前期工作，争取该项目年内开工；做好60万千瓦火力发电厂和年产120万吨水泥厂等重大项目的申报、论证工作，争取早日开工建设，增强全市工业经济的发展后劲。与此同时，还要充分利用市内的资源条件，培育和发展一批新的工业企业。要充分利用我市丰富的农副产品资源优势，发展食品、果蔬等系列食品加工，重点开发罐头食品、保健食品和绿色食品等。利用我市水电资源和矿产资源，按市场要求发展矿产品加工业。发挥我市亚热带林业资源优势，按可持续发展要求，发展林产、林化工业。

　　三、加快推进城镇化，发展城镇经济

　　推进城镇化，要以完善功能、改善环境、提高经济社会承载能力为重点，引导各种要素向城镇聚集，繁荣城镇经济，促进城镇发展。

　　切实搞好城市规划。以建设生态型商贸旅游城市为目标，着重抓好贺州市总体规划调整，按近期、远期的发展规划，把西湾、黄田、鹅塘三镇划入规划区。在此基础上，编制环卫、公交、燃气、排水、旅游、电力、电信、大学园区等专项规划，和太白湖风景区、市体育中心等一批小区规划，以及贺州大道等一批城市街景设计。小区、街景规划设计，要提高设计水准，充分体现我市的功能定位，路、景、灯饰等要与生态型商贸旅游城市定位相衔接。提倡公众参与规划编制，增加规划编制的科学性和透明度。切实加强规划编制、审批和实施的管理，依法确保规划全面、有效实施。年内完成三县一区城镇体系规划。

　　加快城市基础设施建设。积极探索适应市场经济要求的投融资办法，抓好市区道路、通讯、供电、供水、排水、绿化、垃圾污水处理等基础设施建设，重点抓好龟石供水工程、八步城区防洪体系、市垃圾和污水处理厂等一批城建项目；进一步完善教育、文化、广播、电视、体育、医疗、卫生、社会福利等公共服务设施，重点抓好体育中心、图书馆、博物馆、文化艺术中心等一批公共项目的筹建工作，以增强城市的综合服务功能。

　　提高经营城市水平。要引入经营城市理念，把城市的土地、房地产、市政设施、功能要素等作为产业来经营，加大运用市场机制配置土地等资源的力度，努力盘活城市基础设施存量资产，围绕旧城改造、江北东路、太白湖公园、体育中心小区等一批项目建设，通过市场机制，公开招标，在搞好项目配套建设的同时，增加政府税收，有效解决城市建设资金不足的问题，以此推进城市基础设施建设投资主体多元化、投资方式多样化、项目实施市场化。

　　加强市政管理。要依法搞好城市管理，加大综合执法力度，逐步建立城市综合管理的长效机制。做好城市道路维修，搞好城市绿化，特别是要加强对一些出口通道、市区交通干道进行绿化改造。加强城建监察，加大力度对主要街道的监管，拆除市区违章建筑，确保无店外经营、乱摆摊设点、乱搭乱盖等影响市容的违章行为。

　　加大小城镇建设力度。要根据国务院、自治区关于加快小城镇建设的决定，立足我市实际，把发展小城镇作为带动农村经济和社会发展的战略举措，因地制宜完善乡镇基础设施建设，提高城镇服务功能，突出自身特色。要继续以原来确定的公路沿线13个小城镇为重点，开展城镇建设管理“桂东杯”竞赛，进一步搞好城镇规划，加大投入，加强环境综合治理，以此促进小城镇的发展，推进城镇化进程。

　　切实搞好连结城乡的交通建设。配合已开工建设的桂梧高速公路贺州段，做好征地拆迁工作；配合有关部门争取洛湛铁路贺州段尽快开工建设；抓好国道323线莲塘至鹰阳关路段改建工程的前期工作以及富阳至麦岭二级公路改建工程；争取八步至钟山一级公路、昭平三门滩至大爽、八步至公会、八步至姑婆山、昭平至黄村等二级公路立项。加强县（区）级公路和乡村公路、道路运输站场以及港口码头等交通设施建设。

　　四、大力发展以旅游、商贸为主的服务业，培育新的经济增长点

　　旅游业作为我市的一个新兴特色产业，正逐步成为带动第三产业乃至整个经济发展的支柱产业，今年在这方面的工作要有新的突破。在抓好旅游业的同时，适应人流、物流、资金流、信息流等大开放、大发展的市场体系，积极发展商贸流通业和其它服务业，努力为我市经济发展增添新的活力。

发挥资源优势，加快旅游业的发展。今年，要抓紧编制全市旅游总体规划，争取年内完成总体规划的评审工作。采取政府推动、市场引导、企业运作的方式，搞好重点旅游景区景点的规划、开发和建设，特别是临贺故城、昭平七冲原始森林等旅游资源及文物保护项目的规划以及姑婆山二期工程、黄姚古镇后续国债资金项目、贺州石林、客家围屋、紫云洞景区、碧水岩景区、秀水状元村、荷塘十里画廊等景区景点的开发和旅游公路建设，不断提高旅游产品的档次和竞争力。健全旅游行政管理机构，加强旅游行业管理，全面开展整顿和规范旅游市场秩序，改善旅游市场环境，提高旅游服务质量。抓住重点客源市场，采取政府牵头、旅游企业联动以及建立旅游网页等多种方式，实施多元化、全方位旅游宣传促销。在巩固扩大粤港澳客源市场的同时，进一步拓展和开发东南亚国家和海外客源市场。办好下半年在我市召开的全国旅游地理学会第18届年会。抓好旅游发展公司的组建，推进旅游产业化经营。

　　大力发展现代商贸流通业。要在巩固原有商贸流通渠道的基础上，抓紧培育发展一批中小型的商贸流通企业。根据城市消费的特点和变化，积极探索连锁经营等现代流通方式。依据我市地处三省（区）结合部的区位优势，吸引各方投资兴建一批现代流通设施和区域性市场设施，努力打造我市作为桂东地区的重要商贸中心。要加大农村市场开拓力度，进一步搞活农副产品流通，开发适应农村市场需求的产品。加快市场制度和法规建设，强化市场管理与调控，在更大程度上发挥市场在资源配置　中的基础性作用。

　　进一步引导、规范和促进新兴服务业的健康发展。要大力发展房地产业，把房地产业作为加快城镇化的切入点和新的经济增长点来抓，推行小区建设，城市私人建房逐步退出，把居民住宅从分散建设转变为有计划地开发，从而拉动城市建设和相关产业的发展。积极发展邮电通讯、建筑安装和物业管理。加快发展公共交通、教育培训、文化娱乐、卫生保健、体育健身等服务业。进一步发展信息、会计、监理、咨询、法律服务、科技服务等中介服务业。努力发展社区服务业，搞好社区配套服务，完善社区功能。

　　五、强化服务意识，全方位对外开放，大力发展开放型经济和非公有制经济

　　对外开放是我国的一项基本国策，必须坚持国内、国外开放相结合，“引进来”与“走出去”相结合，在更大范围、更宽领域、更高层次上参与国内、国际经济技术合作与竞争，千方百计扩大出口、吸引外资。

　　扩大外贸出口。要以发展农副产品加工品、机电产品为新的出口增长点，在政策服务上实行重点倾斜，促进外贸出口的不断增长。要坚持出口市场多元化战略，积极组织企业参加广交会等各种出口商品交易会，通过制作企业网页，发展电子商务等新的市场开发手段，不断开拓出口市场。要抓住西部大开发的机遇，多争取西部开发资金，提高传统产品的质量、档次和产品附加值，不断调整和优化出口产品结构。要以创造良好的出口环境为中心，努力搞好银贸合作、税贸合作、关贸合作，不断改善出口环境，促进贸易便利化。要用好、用足、用活各种鼓励出口的政策，以政策促进出口。

加大招商引资力度。建立和完善招商引资项目库，以工业、城镇建设、商贸旅游和农副产品加工为重点，筛选一批项目对外进行招商。招商引资既要引进境外资金和技术，也要积极承接东部尤其是广东的产业转移；既要引进劳动密集型企业，也要引进高新技术产业；既要引进大企业，也要引进中小企业。改革招商引资方式，今年要组建招商专业队，以专业队招商为主要形式，举行投资项目推介会，拜访重点客商，洽谈引资项目，促进项目投资。同时还要开展好部门招商、委托招商、网上招商、专题招商、以商引商等多种招商活动，要落实招商责任制和签约项目跟踪责任制，以提高项目招商的质量和成功率。

　　加快发展个体私营等非公有制经济。发展贺州经济，既要充分利用外来资金，也要充分利用本地资金，要把发展个体私营等非公有制经济作为贺州经济发展的一项重要战略任务来抓，毫不动摇地鼓励、支持和引导非公有制经济的发展。要全面落实加快非公有制经济发展的各项政策，优化发展环境，提供优质服务。只要非公有制经济业主能依法经营、照章纳税、保障职工合法权益、符合安全生产和环境保护要求的，一律给予支持，实行国有和民营一视同仁，使非公有制经济成为我市最具活力的经济增长点。

　　进一步改善投资环境。今年，要继续开展“投资环境建设年”活动。要积极营造良好的社会环境、市场环境、法治环境和生活环境，关心、支持和帮助外来企业进行依法管理。要继续推行损害投资环境行为的责任追究制，制止各种损害企业利益的行为。切实治理对企业的“三乱”行为，严格执行“两证一票一卡”收费制度，维护投资者的利益。推行行政服务公示制和行政审批限时制，设立一条龙办证大厅，提高办事效率和质量。各级政府、各部门都要强化服务意识，真心诚意为外商企业和非公有制经济业主排忧解难，责无旁贷地支持好我市外商投资企业和非公制经济发展。与此同时，要进一步整顿和规范市场经济秩序，严厉打击制售假冒伪劣商品的犯罪活动，营造良好的社会信用环境和市场环境。

　　六、加强财源建设，做好增收节支工作

　　今年，我市减收增支因素仍然较多，必须认真贯彻中央、自治区确定的财政税收政策，从紧安排财政支出，千方百计增收节支，努力保持收支平衡。

　　积极培植财源，确保财政稳定增长。要加快推进工业化、城镇化，积极发展机电、建材、冶炼等产业，扶持重点企业的发展，培植支柱财源。调整种养业结构，重点发展松脂、毛竹、烟叶、水果、茶叶等高效农产品，扶持发展农副产品深加工，提高农产品附加值，挖掘农业特产税潜力。大力发展旅游、商贸流通业，培植新兴财源。抓好桂梧高速公路、洛湛铁路贺州段等重点项目的开工建设，增加财政税收。加强现有企业的技改，提高工业企业的整体效益。

　　进一步组织好收入。根据事权和财权相统一的原则，我们对市和八步区的直属纳税业户进行了界定，明确了纳税业户的范围、收入划分等问题，从今年开始，要严格执行。要强化税收征管和税务稽查，坚持依法治税，大力清理各种欠税，做到应收尽收。加强土地有偿使用收入的征管，使其成为非税收入的重要来源。建立激励机制，落实财政税收目标管理责任制，实现财政增收目标。

深化财政改革，建立健全财政管理体制。深化“收支两条线”管理改革，继续将有关部门的预算外收入全部纳入预算管理，实行部门预算内、外资金统管统筹使用的部门综合预算。推进部门预算改革，做好部门预算的试点工作。启动国库集中收付制度改革，逐步扩大财政支付范围。健全工资统发机构，完善制度，扩大统发工资范围。加大政府采购力度，扩大政府采购范围。

　　加强财政支出管理，逐步建立公共财政基本框架。切实加强企业挖潜改造资金、科技专项经费、城市维护建设费等专项资金的管理，并保证资金及时足额到位。进一步规范财政资金供给范围，控制行政经费不合理增长。统筹安排财政资金，优先保证干部职工工资按时发放，确保当年不出现新欠发工资，并努力消化历年欠发工资。严格控制各种一次性追加支出，重新制定和严格执行有关会议费、接待费等各项节约支出的制度。

　　七、努力做好扩大就业和社会保障工作，不断提高人民生活水平

积极扩大就业，改善人民生活，是“三个代表”要求的根本体现，是正确处理改革、发展、稳定关系的着眼点和落脚点，必须从维护改革、发展、稳定大局的政治高度，把扩大就业、改善人民生活的工作抓紧抓好。

　　把就业和再就业工作放在突出位置来抓。要立足于经济发展解决就业问题，积极发展劳动密集型产业，主要是服务业、中小型企业和个体私营经济，重点开发非公有制经济、非农产业和社区家政等新型服务业。认真落实各项就业和再就业扶持政策，加强对就业和再就业工作管理，不断拓展就业空间。进一步做好下岗职工再就业工作，努力提高再就业率。

　　加快完善社会保障体系。要切实把“两个确保”和“一个低保”工作落到实处，做好“两个确保”和“三条保障线”的衔接工作。进一步完善养老、失业、医疗和城镇居民最低生活保障制度，加快形成独立于企业事业单位之外、资金来源多元化、保障制度规范化、管理服务社会化的社会保障体系，建立可靠、稳定的社会保障资金筹措、有效运营和严格管理机制。要进一步扩大社会保险覆盖面，提高资金的征缴率。各县（区）在财政预算中必须优先安排社会保障所需资金，并纳入财政专户管理，保证资金专款专用。要特别关注弱势群体的生活，建立和完善社会救助体系，落实优抚安置政策，切实保障社会特殊群体和特困家庭的基本生活。

　　努力改善城乡居民生活。要在经济发展的基础上改善人民生活，使人民共享经济繁荣成果。坚持“一要吃饭，二要建设”的方针，调整分配结构和支出结构。要千方百计增加农民收入，减轻农民负担，改善农村消费环境，促进农村居民消费增长。加大扶贫力度，帮助贫困群众增加收入，尽快摆脱贫困。抓好城镇基础设施和公共服务设施建设，加强生态和环境治理，改善人居环境，提高城乡居民的生活质量。

　　八、积极发展科技教育和各项社会事业

　　各项社会事业的发展，是加快经济建设的要求，也是社会文明进步的重要标志。要采取切实有效的措施，进一步推动科技、教育、文化、卫生、体育和其他各项社会事业的发展。

加快推进科技创新。以新一轮创新计划为动力，培育和挖掘为产品创新服务的技术源与知识源，加强自主创新和引进消化创新，以强有力的科技支撑，推进工业化、城镇化、信息产业化和农村产业结构调整。扶持和发展一批科技中介服务机构，开展社会化、多样化、专业化服务。组织开展科技、文化、卫生三下乡活动。广泛深入开展科普活动。

　　努力发展各类教育。要深化教师人事制度改革，完善教师全员聘任制。加强师资队伍建设，继续抓好“21世纪园丁工程”的实施，积极开展以骨干教师为重点的全员培训。加强对市直学校的建设和管理，开展全市中小学常规管理年活动。重点发展基础教育，做好扫盲对象的调查摸底和初中控辍工作，巩固和提高“两基”教育成果。发展高中教育，扩大高中招生规模，贺州市高中、昭平中学要抓紧创建自治区示范高中。发展职业教育，将在梧州的四所中等专业学校与市技工学校合并创办贺州职业技术学院。发展高等教育，做好梧州师专大专升本科的申报工作，创办贺州学院。继续办好电大贺州分院与市师范学校联合创建的广西电大远程教育网络贺州分校基地。下力气抓好全市中小学危房改造工作，年内基本消除中小学危房。搞好中小学校的布局调整，优化教育资源配置。

　　大力发展文化、卫生、体育等各项事业。发展先进文化，必须实施精品工程，多出文艺精品；继续推进基层文化建设，巩固和建设好乡镇图书馆和乡镇、村宣传文化站（室），丰富城乡群众文化生活；整顿和规范文化市场秩序，加强网吧、音像市场、娱乐场所和演出市场的管理；实施农村电影放映“2131”工程；加强新闻出版工作，扫黄打非，净化出版物市场和文化市场。加快广播电视网的升级改造，争取在年内建成连接3个县（区）的光缆干线，扩大广播电视无线覆盖面，巩固“村村通广播电视”成果。继续推进农村卫生改革，回建好卫生院危房，组建市疾病预防控制中心、卫生监督所、妇幼保健院和急救中心；以“三网”建设为突破口，认真做好重点传染病的防治工作；加强药品监管行政执法工作，继续推行药品集中招标采购，开展“星级药店”评定活动，完成药品零售企业药品分类管理试点工作，调整农村药品市场批发供应渠道结构，在乡镇一级基本建立仓储式药品配送中心;实施母婴安全工程；搞好农村改厕工作。备战全区第十届运动会，认真筹办市第一届运动会，积极开展以实施全民健身为目标的群众体育活动，努力抓好竞技体育。发展气象、社会福利等事业，做好人民防空、防震减灾工作。关心和支持老龄、妇女、儿童、残疾人等社会事业。

　　切实做好人口、资源管理和环境保护工作。计划生育工作要继续以稳定低生育水平为目标，深入开展“三好一满意”活动，认真落实人口与计划生育目标管理责任制，建立健全计划生育依法管理的运行机制，严格执行“七个不准”。进一步推行计划生育村民自治，到年末实行村民自治的村达到80%。深入开展计生优质服务活动，争取各县（区）都成为先进县。推行环境保护目标责任制，切实加强城市环境综合整治，重点整治交通、金属加工、建筑施工 、修理业、娱乐业的噪声及饮食服务业的烟尘等污染问题。加大城市生活污水的整治和工业污染源的防治力度，特别要加强对小钢厂、小矿山等的监管力度，防止死灰复燃。抓好重点流域、区域的环境保护，主要是搞好饮用水源的保护工作，确保城市饮用水源的安全。加大环境执法力度，严肃查处环境违法行为。严格执行基本农田保护制度，统筹安排好各类建设用地。切实做好国土资源规划和管理，年内基本建立起有形土地市场。

九、加强社会主义精神文明和民主法制建设，为经济和社会发展营造良好环境

　　全面建设小康社会，必须加强精神文明和民主法制建设，坚持依法治国方略和以德治国方针。

努力提高精神文明建设水平。要继续深入学习贯彻党的十六大精神，用十六大精神统领各项工作。以创建桂东文明绿洲为目标，创建文明村镇、文明行业、文明单位，不断深化群众性精神文明创建活动。普及科学知识和科学方法，移风易俗，提倡科学健康文明的生活方式。挖掘我市优秀的民族文化资源，努力创作更多能提高我市影响的文化精品。加强爱国主义教育基地建设，为开展精神文明活动提供有效载体。深入开展“拥军优属、拥政爱民”活动，增进军政、军民团结。搞好全民国防教育，加强国民动员、民兵、预备役工作，发挥工会、共青团、妇联等人民团体和驻贺部队以及民兵在“两个文明”建设中的作用。

　　进一步加强民主法制建设。健全民主制度，保障人民群众的民主权利。各级政府要自觉接受同级人民代表大会及其常委会的监督，自觉接受民主监督、群众监督和舆论监督，积极支持政协工作，主动加强与各民主党派、工商联、无党派爱国人士联系，认真听取各人民团体的意见。加强法制工作，推进政府工作的法制化。进一步做好民族、宗教工作，加强外事、侨办等工作。巩固和扩大基层民主，尤其要抓好村委会换届后制度建设和培训工作，不断完善村民自治管理、社区民主管理和企业民主管理制度，全面推行政务公开、村务公开、厂务公开。深入开展法制宣传教育，普及法律知识，不断增强公民法律意识，提高全体公民特别是领导干部的法律素质。

　　全力做好维护社会稳定工作。以贺州市城区为重点，深入开展“严打”整治专项斗争，大力整治社会治安，重点打击“两抢两盗”以及爆炸、投毒、杀人等严重暴力犯罪，坚决铲除“六合彩”赌博和制、贩、吸毒等社会毒瘤。大力推进“打、防、控”一体化建设，进一步发挥人民调解在社会治安综合治理工作中的第一道防线作用。认真分析新时期的人民内部矛盾，把解决群众的实际问题作为重点和关键环节，深入做好思想政治工作，认真抓好矛盾纠纷尤其是土地、水利和山林“三大纠纷”的排查调处，妥善处理群体性事件，有效化解矛盾。加强国家安全保密工作。坚持“安全第一，预防为主”的方针，继续以矿山、危险化学品、易燃易爆品、公共聚集场所消防、道路交通和农机安全生产为重点，开展安全生产专项整治，完善安全生产设施，强化安全生产管理和安全监察，严格执行安全　生产责任制，避免重大安全事故发生。

　　十、加强和改进政府工作，提高政府管理经济和社会事业水平

　　新的形势和任务，对政府工作提出了更高的要求，我们要以奋发有为、与时俱进的精神状态，按照市委提出的“迎难而上、团结奋进、热爱贺州、真诚奉献”的贺州精神的要求，切实加强政府自身建设，推进经济建设和各项社会事业的发展。

　　转变政府职能。适应撤地设市后的新要求，加快建立与市场经济体制相适应、与市一级管理权限和职责相匹配的办事高效、运转协调、行为规范的行政管理体制，重点实现两个转变：一是实现由过去地区一级的行政体制和管理职能向市一级行政体制和管理职能转变；二是实现由过去地区一级工作方式和思维方式向市一级的工作方式和思维方式转变。在此基础上，按照建立社会主义市场经济体制的要求，实行政企、政事分开，切实把政府的职能转到经济调节、市场监管、社会管理和公共事务上来，减少对微观经济活动的直接干预，充分发挥市场配置资源的基础性作用。继续深化行政审批制度改革，大力精简审批事项和简化审批程序；公开国民经济与社会发展计划、收费、许可、政府采购等行政决策事项，提高透明度；坚持权利与责任相统一，实行审批责任追究；建立行政审批监督、制约机制，防止以权谋私。

　　严格依法行政。政府工作人员要进一步增强依法行政的观念，认真学习法律、法规和世贸组织规则，学会运用法律手段管理经济、文化与社会事务。要严格执行国家和自治区制定的各项法律、法规及行政规章，确保法律、法规的权威和政令的统一。完善政府规章和其他行政措施，坚持统一、公正、合理地行政执法。重视对行政规章和其他政策措施的备案审查工作，提高行政执法的透明度，推动政府管理行为的规范化、法制化。强化执法监督，推行执法责任制、行政管理公示制、评议考核制，规范各级政府和政府各部门及其工作人员的行政行为，督促各部门领导干部带头依法行政，做到有权必有责、用权受监督、侵权要赔偿。

　　改进工作作风。各级政府要以“三个代表”思想统揽全局，指导工作，用创新的思维去谋划工作，开展工作，更好地适应新形势、新任务的要求。要深入基层、深入群众，倾听群众呼声，关心群众疾苦，为群众解决实际问题。要狠抓各项工作的落实，严格执行“机关岗位经济目标考核责任制”，切实做到人人有目标，事事有落实。各部门要充分发挥职能作用，改变推诿扯皮、应付了事的坏习气。加强政府同社会各界的沟通，尤其是要认真听取人大代表、政协委员的意见，不断提高决策的民主化、科学化水平。坚决反对官僚主义和形式主义，进一步密切政府与人民群众的血肉联系。

坚持勤政廉政。要认真贯彻胡锦涛总书记在河北西柏坡考察时提出的“两个务必”的要求，大力弘扬谦虚谨慎、不骄不躁和艰苦奋斗的作风。严格执行中央、自治区、市有关廉政建设的规定，厉行节约，坚决遏制用公款大吃大喝、游山玩水和高消费娱乐。要加强对政府各部门工作人员特别是领导干部的理想信念和廉洁从政教育，增强自我约束能力，做廉洁自律的表率。要健全制度，完善监督制约机制，严肃财经纪律，强化依法审计，从源头上、机制上、制度上预防和治理腐败。切实纠正部门和行业不正之风，加大查处违法违纪案件的力度，对以权谋私、贪赃枉法、行贿受贿的人和事，坚决查处，决不姑息。

　　各位代表，当前国内外形势正发生着深刻的变化。在新的形势下，我们面对着许多历史性机遇，我市经济社会发展的潜力很大，前景很好。让我们紧密团结在以胡锦涛同志为总书记的党中央周围，高举邓小平理论伟大旗帜，深入贯彻“三个代表”重要思想，在市委领导下，解放思想，与时俱进，同心同德，扎实工作，为全面完成今年我市经济和社会发展各项任务而努力奋斗！
